# New Assessment Model of Pulse Depth Based on Sensor Displacement in Pulse Diagnostic Devices

**DOI:** 10.1155/2013/938641

**Published:** 2013-09-26

**Authors:** Jang-Han Bae, Young Ju Jeon, Jong Yeol Kim, Jaeuk U. Kim

**Affiliations:** Biomedical Engineering Research Division, Korea Institute of Oriental Medicine, Daejeon 305-811, Republic of Korea

## Abstract

An accurate assessment of the pulse depth in pulse diagnosis is vital to determine the floating and sunken pulse qualities (PQs), which are two of the four most basic PQs. In this work, we proposed a novel model of assessing the pulse depth based on sensor displacement (SD) normal to the skin surface and compared this model with two previous models which assessed the pulse depth using contact pressure (CP). In contrast to conventional stepwise CP variation tonometry, we applied a continuously evolving tonometric mechanism at a constant velocity and defined the pulse depth index as the optimal SD where the largest pulse amplitude was observed. By calculating the pulse depth index for 18 volunteers, we showed that the pulse was deepest at *Cheok* (significance level: *P* < 0.01), while no significant difference was found between *Chon* and *Gwan*. In contrast, the two CP-based models estimated that the pulse was shallowest at *Gwan *(*P* < 0.05). For the repeated measures, the new SD-based model showed a smaller coefficient of variation (CV ≈ 7.6%) than the two CP-based models (CV ≈ 13.5% and 12.3%, resp.). The SD-based pulse depth assessment is not sensitive to the complex geometry around the palpation locations and temperature variation of contact sensors, which allows cost-effective sensor technology.

## 1. Introduction

Pulse characteristics are generally influenced by vascular compliance, blood viscosity, and the overall function of major organs [1]. Pulse signals measured from *Chon (Cun), Gwan (Guan), *and* Cheok (Chi)*, which are three adjacent regions along the radial artery used for pulse diagnosis in traditional Eastern medicine (TEM), can reflect a patient's physical condition and pathological problems [2]. Therefore, pulse diagnosis has been considered an important diagnostic method in TEM for thousands of years.

Pulse diagnosis requires a high level of palpation skill and long-term experience; a historical medical doctor, Shuhe Wang, mentioned in his book, *The Pulse Classic* (*Mai Jing*), that an experienced physician can easily understand a patient's holistic physical condition using pulse diagnosis, but the technique is limited because fingertip sensations are difficult to articulate [3]. As long as pulse diagnosis remains in the realm of fingertip-reading, it will be a difficult skill to master and have a great deal of subjectivity in interpretation.

Recently, a number of scientific achievements have been made towards quantification of pulse diagnosis [4]. For example, advances in sensor technology have shifted singleelement sensors to multielement sensors, providing more accurate and diverse information on the pressure pulse wave (PPW), including spatial information using strain gauge, piezoresistor, or polyvinylidene fluoride (PVDF) [5–7]. Using clinical studies and statistical methods, numerous classification models have been proposed to quantify principal pulse qualities (PQs), including the floating, sunken, deficient, excess, moderate, smooth, taut, hollow, and unsmooth PQs [1, 8–15]. Further progress has been made in pulse-signal processing and noise reduction [16–20]. In addition, studies verifying pulse characteristics of palpitation patients [21], dyspepsia and rhinitis patients [22], and cold/heat-stressed humans [23] and studies explicitly comparing pulse characteristics among the three pulse-diagnostic locations [24–26] were recently reported. All of these research activities will move up the development of objective and reliable pulse diagnostic devices.

Here, we revisit the issue of classifying the floating and sunken PQs and discuss how to assess the pulse depth. Correct assessment of the pulse depth is vital in diagnosing the pulse [27]; the floating and sunken PQs are two of the four most basic PQs (with the rapid and slow PQs as the other two). Moreover, the pulse depth is needed to correctly identify approximately one third of the 28 classical PQs including the hidden, string, faint, weak, deficient, soft, scattered, surging, and scallion-stalk PQs [2, 28].

Thus far, two competing classification models for the floating and sunken PQs have been proposed [8, 15], both of which assessed the pulse depth by tracing the pulse amplitude variation as a function of contact pressure (CP). In this work, we propose a new pulse depth assessment method that traces the pulse amplitude variation as a function of sensor displacement (SD) in the direction normal to the skin surface. We show that the SD-based pulse depth assessment is qualitatively distinct from the CP-based methods, and it yields improved repeatability and clinically desirable pulse depth distribution among *Chon*, *Gwan*, and *Cheok*.

## 2. Pulse Depth Assessment

### 2.1. Literature Review

A desirable pulse depth assessment method should be useful in classifying the floating and sunken PQs. Therefore, a clear understanding of the floating and sunken PQs is essential prior to discussing the pulse depth assessment. In the following, we compare representative expression for the floating and sunken pulses appeared in pulse classical literature.

According to *The Pulse Classic (Mai Jing)*, the floating pulse is a pulse felt potent with no force applied on the fingertip but felt impotent with force applied, and the sunken pulse is a pulse felt impotent with no force applied on the fingertip but felt potent with force applied [3, 29]. The *Difficult Classic (Nan Jing)* states that the floating pulse flows above the muscle layer [30, 31]. According to *The Lakeside Master's Study of the Pulse (Bin Hu Mai Xue)*, the floating pulse has power in the pulsation when fingertip touches lightly on the skin layer and is felt powerless when pressed down, and the sunken pulse is a pulse that can be felt when the fingertip is pressed down to the musculoskeletal level [32]. In the *Elementary Course for Medicine (Yi Xue Ru Men)*, the floating pulse has shortage of strength when fingertip is applied strongly, but it has surplus of strength when fingertip is applied slightly, and Sunken pulse has shortage of strength when fingertip is applied slightly, but it has surplus of strength when relatively strong fingertip is applied [33]. 

In summary, the floating pulse is a shallow-lying pulse, and the sunken pulse is a deep-lying pulse. Here, we notice that “shallow-lying” or “deep-lying” can be estimated either in terms of CP or SD. To the best of our knowledge, all recent studies assessing the pulse depth did so by tracing the pulse amplitude variation as a function of CP [2, 8, 14, 15]. As it will be discussed below, tracing the pulse amplitude as a function of SD is qualitatively distinct from CP-based methods. After introducing two competing CP-based pulse depth assessment methods, we propose a new SD-based method. 

### 2.2. Contact Pressure as an Assessment of Pulse Depth

**Figure 1:**
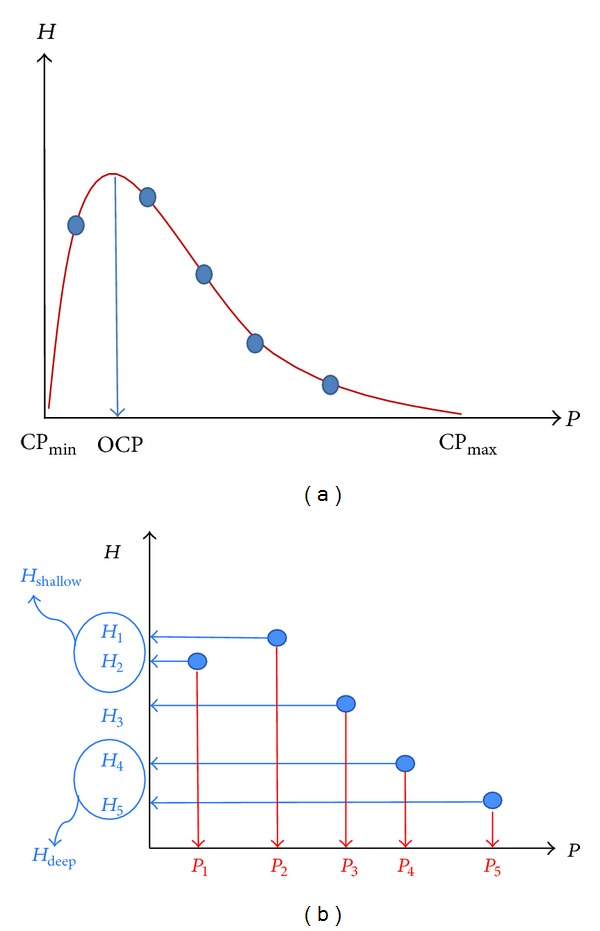
Two CP-based models to assess the pulse depth. (a) CFS_ocp_ is an index to assess the pulse depth based on the magnitude of the optimal contact pressure (OCP) when the maximum pulse amplitude is obtained. (b) CFS_pad_ is an index to assess the pulse depth by the explicit difference between *H*_deep_ (pulse amplitude at a high CP) and *H*_shallow_ (pulse amplitude at a low CP).

The first step in assessing pulse depth using CP is to obtain the pulse amplitude as a function of CP, which is called the *P-H* curve as coined by Fei [2]. Using the *P-H* curve, two competing models were proposed to assess the pulse depth; one can estimate the optimal contact pressure (OCP) where the pulse amplitude reaches its maximum, and the other one can estimate the difference of pulse amplitudes at a high CP and low CP (see Figure 1).

In the first model, the OCP was estimated and normalized by the range of applied CP [15];

(1) $$\begin{array}{c} {\text{CFS}}_{\text{ocp}}=\frac{\text{OCP}-{\text{CP}}_{\min}}{{\text{CP}}_{\max}-{\text{CP}}_{\min}}, \end{array}$$

where CP_max⁡_ is the maximum applied CP and CP_min⁡_ is the minimum applied CP. Here, CFS stands for the coefficient of the Floating and Sunken pulses, and CFS_ocp_ is the CFS based on the OCP estimation. CFS_ocp_ is bounded between 0 and 1, and it approaches 0 (1) as the pulse amplitude reaches its maximum at a low (high) CP. Therefore, a small CFS_ocp_ and OCP (a large CFS_ocp_ and OCP) are indicative of the floating-like (sunken-like) pulse. Ideally, the minimum CP is zero (no contact pressure applied), and the maximum CP is a large constant that covers the blood pressure range of the majority of the population. Under these ideal conditions, the CFS_ocp_ is directly proportional to the OCP.

The second model compares the pulse amplitude at a small CP with that at a large CP [8];

(2) CFSpad=12(1+Hdeep−HshallowHdeep+Hshallow)=HdeepHdeep+Hshallow,

where *H*_deep_ is the pulse amplitude at a large CP, *H*_shallow_ is the pulse amplitude at a small CP, and CFS_pad_ is the CFS calculated by the pulse amplitude difference. According to (2), CFS_pad_ is defined as the difference between *H*_deep_ and *H*_shallow_; it is bounded between 0 and 1, and it approaches 1 (0) as *H*_deep_ (*H*_shallow_) grows larger than *H*_shallow_ (*H*_deep_).

Figure 1 depicts the two CP-based models to assess the pulse depth. To obtain the pulse data, a pulse-taking device measured the pulse signals at five different CP steps (at approximate CPs of 40, 70, 110, 140, and 180 mmHg) for 5 seconds at each step; the amplitudes are presented as filled circles. As shown in Figure 1(a), a continuous *P-H* curve connecting the five distinct *P-H* data points is required to obtain an accurate estimation of the OCP (a cubic interpolation technique was employed in the figure). However, interpolation is not needed to calculate CFS_pad_, as shown in Figure 1(b), leading to a simpler and more robust model in regards to data anomaly [8].

### 2.3. Sensor Displacement as an Assessment of Pulse Depth

To measure a pulse, a pulse-reading sensor was placed normal to the skin surface at a palpation location (PL) and forced down towards the artery to acquire the responsive PPW signal. In this tonometric process, the CP on the detection sensor increases monotonically with increasing SD. At small SDs, the response of the CP is linear. However, as we will discuss more detail in the discussion section, at larger SDs, the response of the CP becomes nonlinear, eventually diverging sharply near the artery occlusion. Because of this nonlinear relationship between the SD and CP, the SD-based pulse depth assessment method may offer qualitatively distinct features from CP-based methods. 

**Figure 2:**
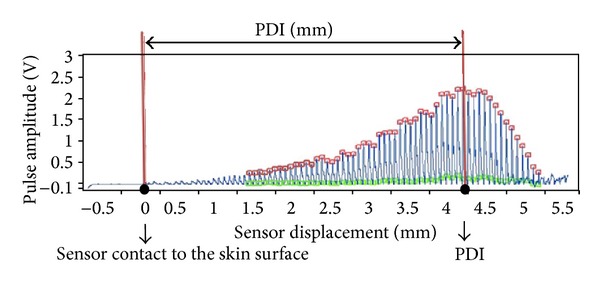
The pulse signal obtained with a CETM and the definition of the PDI. We took the skin contact point as the reference point (*x* = 0).

In this work, we introduce the concept of the pulse depth index (PDI) as a measure of the pulse depth based on the actual SD in the direction normal to the skin surface. The PDI is defined as the sensor displacement from the point of contact with the skin surface to the optimal sensor point towards the radial artery at which the maximum pulse amplitude is observed (see Figure 2).

As shown in Figure 2, to measure the pulse signal using the SD, we adopted a continuously evolving tonometric mechanism (CETM) [34]. The CETM provides a continuous spectrum of the PPW with respect to the SD or CP; therefore, it does not require interpolation to obtain the *P-H* curve or perform further data processing. A detailed discussion of the benefits of the CETM will be discussed later. We show that the SD-based pulse depth estimation is more repeatable and stable.

## 3. Subjects and Methods

### 3.1. Study Subjects

We acquired the tonometric pulse signals at three PLs on the left wrist twice for 18 volunteer subjects, incorporating both males and females. Subjects were in a comfortable upright position during pulse measurements. The study was approved by the ethics committees of the Korean Institute of Oriental Medicine, and informed written consent for the study was obtained from all subjects prior to study entry (I0903-01-02). Basic physiological subject data are summarized in Table 1.

### 3.2. Palpation Locations of *Chon*, *Gwan*, and *Cheok*

The PLs used for pulse diagnosis have changed throughout history. In modern times, three adjacent locations along the radial artery in both wrists have been used, called *Chon, Gwan*, and* Cheok*. The pulse characteristics at *Chon* describe the function of organs located in the upper region of the trunk and the thoracic cavity, such as the lung and heart. Similarly, the pulse characteristics at *Gwan* and *Cheok* describe the upper abdominal cavity (liver, spleen, and pancreas) and lower abdominal cavity (urinary and reproductive organs), respectively [24]. To diagnose the pulse, practitioners put the index, middle, and ring fingers on *Chon*, *Gwan*, and *Cheok*, respectively, and determine the PQs by applying various forces.

**Figure 3:**
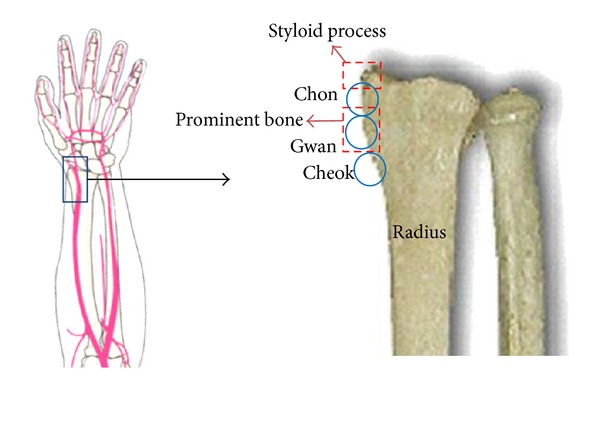
Illustration of the palpation locations of *Chon, Gwan*, and *Cheok.*

The anatomical landmark used to determine the locations of *Chon, Gwan*, and* Cheok* is the prominent bone. Sometimes confused with the styloid process, the prominent bone is a prominentia of the skeleton which is slightly more proximal than the styloid process [35]. Exact locations of the three PLs vary with the patient's elbow-length. A clinical study on 200 adult subjects showed that the lengths of *Chon*, *Gwan*, and *Cheok* were approximately 6%, 6%, and 7% of the elbow-length, respectively, in accordance with ancient TEM records [36]. In particular, 6% of the elbow-length was approximately 1.54 cm for males and 1.4 cm for females due to differing elbow-lengths between male and female subjects. In another recent survey on 78 adult subjects, *Chon* was approximately 1.14 cm distal to *Gwan*, and *Cheok* was approximately 1.49 cm proximal to *Gwan* [35]. As shown in Figure 3, we standardized the three PLs by defining the region between the styloid process and the prominent bone as *Chon*, the region around the prominent bone as *Gwan* that is approximately 10 mm proximal to *Chon*, and the region approximately 13 mm proximal to *Gwan* as *Cheok *[30–32].

### 3.3. Pulse Tonometric Device

**Figure 4:**
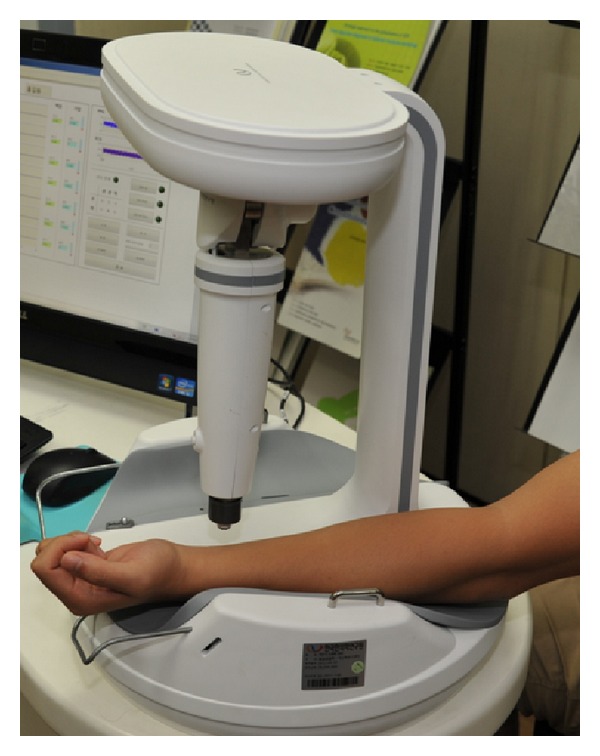
The pulse tonometric device and illustration of pulse signal measurement.

The PPW was acquired using a pulse tonometric device that was developed at the Korean Institute of Oriental Medicine. This device consists of a main body with an arm holder and a sensing body attached to a mobile actuator. A pulse detection sensor, which is composed of 7 piezoresistive unit sensors within 9 × 9 mm^2^, is located at the actuator tip. To measure the PPW, we used the CETM; after locating the sensor at each PL, the device reads the responsive pulse signal, while the actuator steadily moves towards the pulsatile artery at 0.09 mm/sec. By multiplying the elapsed time by the velocity of the moving sensor, we can obtain the sensor displacement. An example of using the pulse tonometric device to measure a pulse is illustrated in Figure 4, and the resulting PPW with respect to the SD is shown in Figure 2. A trained operator measured the pulse signals sequentially in the order of *Gwan*, *Chon*, and *Cheok*. The signal detection sampling rate was 200 Hz. 

### 3.4. Signal Processing and Analysis Methods

Raw data contained noise and baseline wander caused by breathing, subtle movement of subject's arm, and so forth, during pulse measurement. To remove noise and baseline wander, we used a band-pass filter, FFT analysis, and the spline interpolation technique. To find the initiation time of every upstroke, we employed the intersecting tangent method [32, 37]. 

To calculate the PDI, we developed an automated algorithm to detect the contact point of the sensor to the skin surface and the optimal sensor displacement at which the maximum pulse amplitude was observed. For comparison, we also calculated pulse depths using the CP-based models. First, we calculated the OCP by reading the CP value at the optimal sensor displacement to estimate CFS_ocp_; because CFS_ocp_ is linearly proportional to the OCP, we used the calculated OCP in the first CP-based model. Second, to calculate CFS_pad_, we segmented the CPs ranging from 60 mmHg to 195 mmHg into five CP sectors. We calculated the differences between the pulse amplitudes at discrete pairs of high and low CPs to obtain the CFS_pad_.

## 4. Results

For the data analysis, we focused on the statistical differences of the pulse depth distribution among the three PLs. We did not observe statistical differences between male and female subjects, and thus the gender difference was not taken into account.

Table 2 shows the comparison of OCP, CFS_pad_, and PDI with their coefficient of variations (CVs) for the participants. The mean of OCP was estimated to be 134.03 mmHg, 96.03 mmHg, and 134.61 mmHg for *Chon, Gwan*,and* Cheok*, respectively. The OCP was noticeably smaller at *Gwan* than at *Chon* or *Cheok*, which implies that the pulse at *Gwan* was the shallowest among the three PLs. A similar result was reported previously with a larger population group [26]; in terms of OCP, the pulse was shown to be shallowest at *Gwan* and deepest at *Cheok* for 213 female subjects in their 20s and 174 female subjects in their 60s. Likewise, the mean of CFS_pad_ was estimated to be smallest at *Gwan* (CFS_pad_ = 0.41) and larger at *Chon* (CFS_pad_ = 0.52) and *Cheok *(CFS_pad_ = 0.51). In contrast to the CP methods, in the new SD-based pulse depth estimation, the PDI was estimated to be deepest at *Cheok* (PDI = 5.01 mm), and the pulse depth at *Chon* (PDI = 3.76 mm) was comparable to that at *Gwan* (PDI = 3.60 mm).

According to a recent report, the average artery depth measured by ultrasonography for 44 adult subjects was approximately estimated to be 5.1 mm (skin layer above the artery *≈*2.4 mm, artery thickness *≈*2.7 mm) [25]. Taking into account soft tissue below the artery (especially at *Cheok*), artery may be moved down for some distance (a few millimeters) with downward force, and the PDI reaching 5.0 mm is within the reasonable estimation range. In this study, obese subjects were observed with higher value of PDI and CP compared with thin subjects at all PLs, which are in accordance with previous studies [15] and reflect that both PDI and CP are proportional to the wrist thickness.

In regards to the repeatability of the three models, PDI showed a reduced coefficient of variation (CV = 6.55% for *Chon, *6.08% for *Gwan, *10.24% for *Cheok*) compared with OCP (*Chon*: 12.57%, *Gwan*: 11.31%, *Cheok*: 13.12%) and CFS_pad_ (*Chon*: 16.17%, *Gwan*: 12.41%, *Cheok*: 11.93%). This result indicates that, among the three pulse depth estimation models, the PDI-based estimation was the most stable in terms of repeatability. 

**Figure 5:**
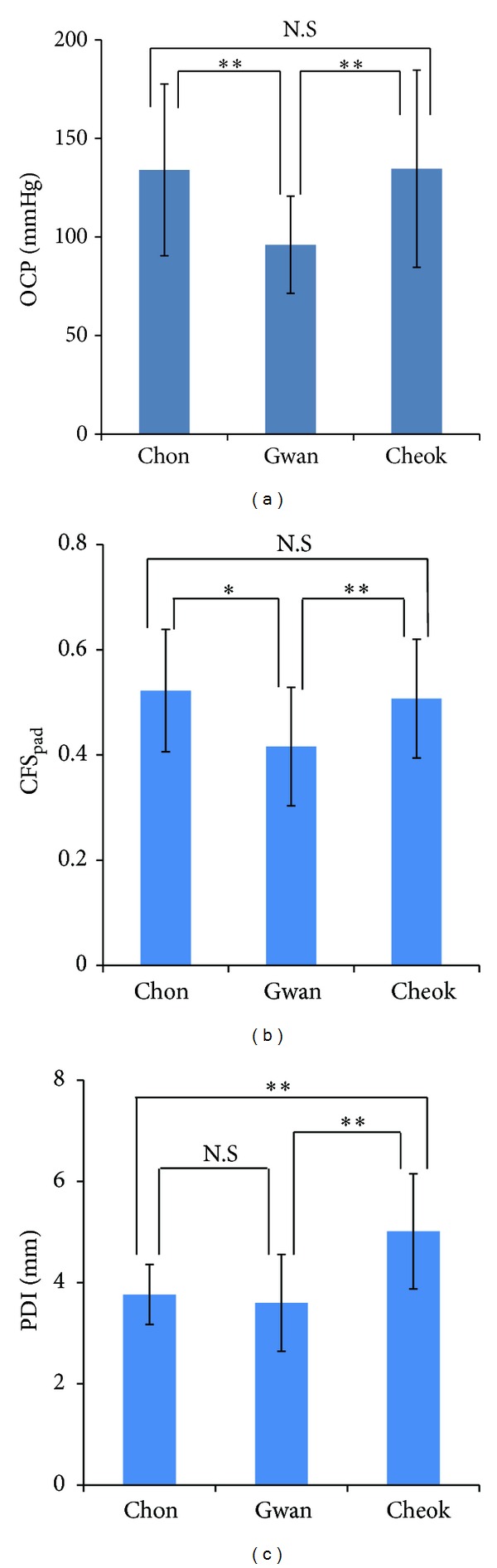
The mean differences of the pulse depth across *Chon, Gwan*, and *Cheok* with Duncan's post hoc test, estimated by (a) OCP, (b) CFS_pad_, and (c) PDI models. *P < 0.05, **P < 0.01, N.S.: not significantly different.

To more quantitatively examine the mean differences between the pulse depths at the three PLs, we performed one-way analysis of variance (ANOVA) and Duncan's post hoc test.  Figure 5 shows the mean differences of the pulse depth at the three PLs with Duncan's post hoc test; both OCP and CFS_pad_ were smallest (i.e., the pulse was shallowest) at *Gwan* (*P* < 0.05), and there was no significant difference between *Chon* and *Cheok*. In contrast, the PDI was largest (the pulse was deepest) at *Cheok* (*P* < 0.01), and no significant difference was found between *Chon* and *Gwan*. 

**Figure 6:**
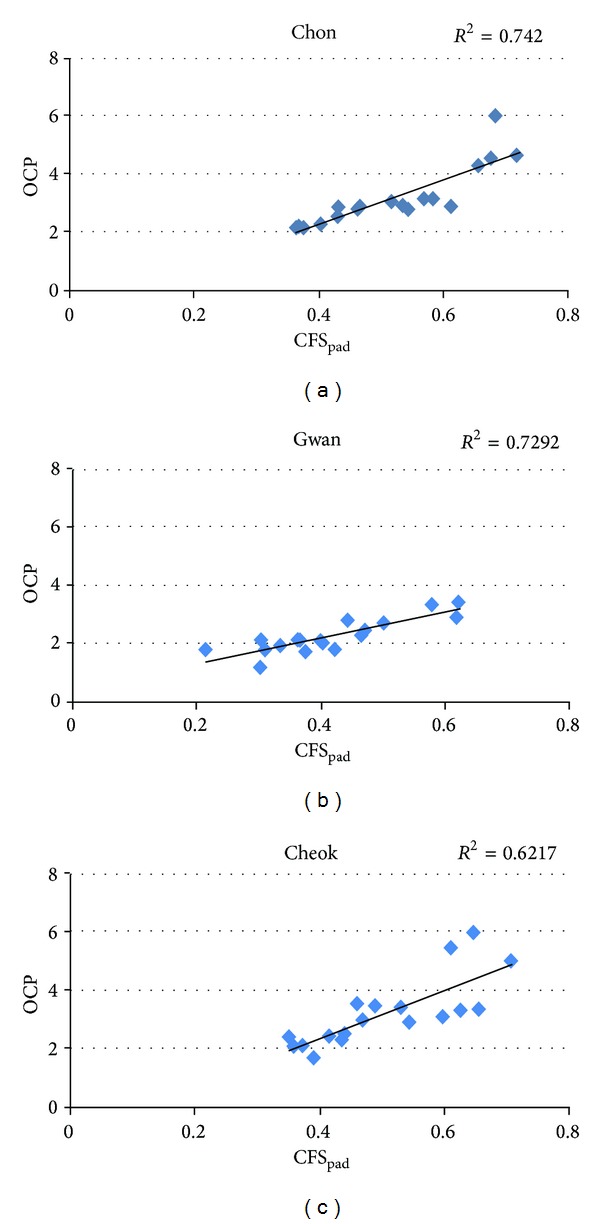
Correlation analysis and the coefficient of determination (*R* ^2^) between the CFS_pad_ and OCP, obtained at (a) *Chon*, (b) *Gwan*, and (c) *Cheok*.

**Figure 7:**
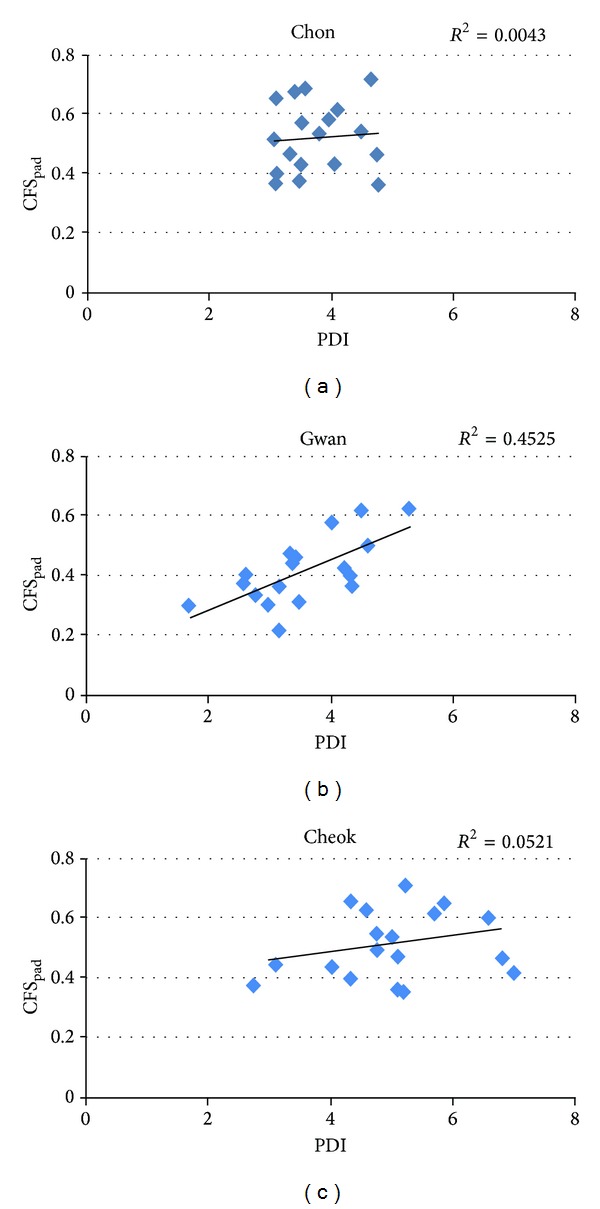
Correlation analysis and the coefficient of determination between the PDI and CFS_pad_ obtained at (a) *Chon*, (b) *Gwan*, and (c) *Cheok*.

For a more detailed comparison between the new PDI model and the two CP-based models, we performed a correlation analysis. As shown in Figure 6, the pulse depths estimated by the two CP-based models, that is, OCP and CFS_pad_, were strongly correlated at all the PLs; the coefficients of determination were *R* ^2^ = 0.74 at *Chon*, *R* ^2^ = 0.72 at *Gwan*, and *R* ^2^ = 0.62 at *Cheok*. In contrast, the PDI and CFS_pad_ showed no correlation at *Chon* (*R* ^2^ = 0.01) or *Cheok* (*R* ^2^ = 0.05) and a reduced correlation at *Gwan* (*R* ^2^ = 0.45), as shown in Figure 7. Likewise, no correlation was found between the PDI and OCP at *Chon* (*R* ^2^ = 0.01) and *Cheok* (*R* ^2^=0.14), and a reduced correlation was found at *Gwan *(*R* ^2^ = 0.49).

Results from the analysis of mean differences and the correlation analysis indicate that the new SD-based and the two previous CP-based pulse depth estimations are qualitatively different, whereas a strong correlation was found between the two CP-based methods. The qualitative difference between the SD-based and CP-based pulse depth estimations is due to the intrinsic nonlinear relationship between the SD and CP. At small SDs, the skin and artery have elastic responses, and the CP (and equivalently force) is linearly proportional to the SD. However, the CP and SD become power-law related at larger SDs. Finally, at the largest SDs, where the artery is close to occlusion, CP diverges sharply with respect to SD, and the power-law relation becomes no longer valid. In this largest SD regime, relevant pulse signals that contain clinical information are not obtainable. 

**Figure 8:**
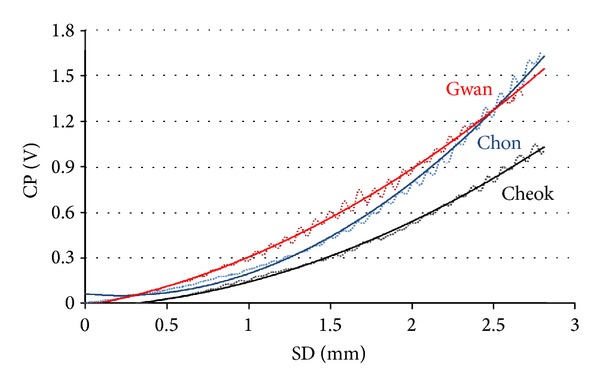
Functional relationship between the CP and SD. Raw data (dots) are features averaged over 18 subjects at each PL, and the quadratic polynomial approximations are plotted in solid curves. In descending order of CP at SD = 2 mm, the data measured are *Gwan* (top, red), *Chon* (middle, blue), and *Cheok* (bottom, black).

Figure 8 depicts the functional relationship between the CP and SD measured at *Chon* (middle, blue), *Gwan* (top, red), and *Cheok* (bottom, black). The raw data (dots) present average features of 18 subjects at each PL, and the solid curves present the quadratic polynomial approximations. The graph clearly shows an initial linear relationship at small SDs and power-law behavior (with an approximate power-law exponent of 2) at large SDs. Indeed, the power-law exponent at *Gwan* is below 2, at *Cheok* slightly above 2, and at *Chon* larger than that at *Cheok*. These values were found by comparing the quadratic approximation curves of the raw data in Figure 8. We found the same results when we applied linear regression; the coefficients of determination were* R* ^2^ = 0.97 at *Gwan, R* ^2^ = 0.95 at *Check, *and *R* ^2^ = 0.91 at *Chon. *This nonlinear relationship between the SD and CP and slightly different power-law exponents among the PLs that are the reasons of no correlation (*Chon* and *Cheok*) or reduced correlation (*Gwan*) between the SD-based and CP-based pulse depth estimations were observed. The sharply diverging relationship between the CP and SD occurred at larger SDs. (not presented in Figure 8).

## 5. Discussion and Conclusions

Classification models for primary pulse qualities (PQs) have recently been developed to meet increasing requests for reliable pulse tonometric devices [1, 8, 11–15]. An important achievement of such efforts was the development of two clinically verified classification models for the floating and sunken PQs, both of which are based on the pulse amplitude variation as a function of contact pressure (CP). In this work, we proposed a novel assessment model of the pulse depth based on actual sensor displacement (SD) rather than CP. Using a clinical study with 18 volunteers, we showed that the proposed SD-based pulse depth assessment was qualitatively distinct from the previous CP-based models and illustrated that this qualitative difference originates from the nonlinear relationship between the SD and CP, especially at large SDs near the artery occlusion. Finally, we showed that the new SD-based model was more repeatable and that the distribution of the mean pulse depths among the three PLs was more in line with clinical experiences than the CP-based models (Clinical consensus is that the pulse depth at *Cheok* is deepest irrespective of gender or age. On the other hand, the pulse depth at *Chon* is disputably shallowest. Our data shows no clear distinction in the PDI between *Chon* and *Gwan*, and we would like to postpone the conclusion to a later work with more extensive clinical study.) 

The radial artery at PLs is approximately 2.5 ± 1.0 mm in diameter, and tendons, muscles, and bony structures are located within ±5 mm from the artery [25, 38], which make it difficult to accurately assess CP applied on the radial artery. In addition, sensor temperature may vary a few degrees during pulse measurement due to contact to the skin surface. The temperature variation (Δ*T*) leads to the time-varying baseline shift (Δ*B*), as it was reported that Δ*B≈*25 mmHg for Δ*T* = 10°C for a piezoresistive pressure sensor [7]. These anatomical complications and temperature dependence of contact sensors are major causes for an erroneous estimation of CP. In contrast, the SD measurement is not sensitive to these complicated geometry or thermal variations. Note that the pulse signal estimation is little affected by nearby obstacles or by temperature variations. Therefore, the proposed SD-based pulse depth assessment model is less erroneous than the CP-based models, and it is applicable to broader types of pulse tonometric sensors. 

In conclusion, we proposed a novel assessment model of the pulse depth based on SD. We showed that the new SD-based pulse depth assessment model is more repeatable and less erroneous than CP-based models, because the SD model yields a robust data against complicated geometric structure around the prominent bone of both wrists and temperature dependence of contact sensors. It remains for further verification if the proposed SD-based pulse depth assessment model outperforms the CP-based models in the aspect of clinical validity. In connection with [39], we find that the SD-based pulse depth assessment is more appropriate than the CP-based methods in the decision of Fu (shallow), Zhong (middle), Chen (deep) levels. The new SD-based pulse depth assessment model with improved repeatability and transparent interpretation will be beneficial in developing contemporary pulse tonometric technology.

## Figures and Tables

**Table 1:** Subject information.

Characteristic (unit)	Value (mean ± SD)
Number (*n*)	18 (male = 8, female = 10)
Age (yr)	58.3 ± 12.2
Height (cm)	158.1 ± 7.8
Weight (kg)	58.16 ± 8.9
BMI (kg/m^2^)	23.2 ± 2.6
Systolic/diastolic blood pressure (mmHg)	119.6/77.3 ± 11.4/9.2

**Table 2:** Comparison between CP-based and SD-based pulse-depth estimations at the three PLs. Data presented are the mean ± SD and CVs for two repeated measures.

PL	Index					
	OCP (mmHg)		CFS_pad_		PDI (mm)	
	mean ± SD	CV (%)	mean ± SD	CV (%)	mean ± SD	CV (%)
*Chon *	134.03 ± 43.57	12.57	0.52 ± 0.11	16.17	3.76 ± 0.59	6.55
*Gwan *	96.03 ± 24.61	11.31	0.41 ± 0.11	12.41	3.60 ± 0.95	6.08
*Cheok *	134.61 ± 50.01	13.12	0.51 ± 0.11	11.93	5.01 ± 1.13	10.24
